# Optimized Prime Editing of Human Induced Pluripotent Stem Cells to Efficiently Generate Isogenic Models of Mendelian Diseases

**DOI:** 10.3390/ijms26010114

**Published:** 2024-12-26

**Authors:** Rodrigo Cerna-Chavez, Alba Ortega-Gasco, Hafiz Muhammad Azhar Baig, Nathan Ehrenreich, Thibaud Metais, Michael J. Scandura, Kinga Bujakowska, Eric A. Pierce, Marcela Garita-Hernandez

**Affiliations:** Ocular Genomics Institute, Massachusetts Eye and Ear Infirmary, Department of Ophthalmology, Harvard Medical School, Boston, MA 02114, USA; rcerna-chavez@meei.harvard.edu (R.C.-C.); albaortega@ub.edu (A.O.-G.); hmbaig@meei.harvard.edu (H.M.A.B.); nehrenreich@meei.harvard.edu (N.E.); tmetais@meei.harvard.edu (T.M.); michael.scandura@yale.edu (M.J.S.); kinga_bujakowska@meei.harvard.edu (K.B.); eric_pierce@meei.harvard.edu (E.A.P.)

**Keywords:** prime editing, hiPSCs, IRDs, isogenic models, editing efficiency

## Abstract

Prime editing (PE) is a CRISPR-based tool for genome engineering that can be applied to generate human induced pluripotent stem cell (hiPSC)-based disease models. PE technology safely introduces point mutations, small insertions, and deletions (indels) into the genome. It uses a Cas9-nickase (nCas9) fused to a reverse transcriptase (RT) as an editor and a PE guide RNA (pegRNA), which introduces the desired edit with great precision without creating double-strand breaks (DSBs). PE leads to minimal off-targets or indels when introducing single-strand breaks (SSB) in the DNA. Low efficiency can be an obstacle to its use in hiPSCs, especially when the genetic context precludes the screening of multiple pegRNAs, and other strategies must be employed to achieve the desired edit. We developed a PE platform to efficiently generate isogenic models of Mendelian disorders. We introduced the c.25G>A (p.V9M) mutation in the *NMNAT1* gene with over 25% efficiency by optimizing the PE workflow. Using our optimized system, we generated other isogenic models of inherited retinal diseases (IRDs), including the c.1481C>T (p.T494M) mutation in *PRPF3* and the c.6926A>C (p.H2309P) mutation in *PRPF8*. We modified several determinants of the hiPSC PE procedure, such as plasmid concentrations, PE component ratios, and delivery method settings, showing that our improved workflow increased the hiPSC editing efficiency.

## 1. Introduction

Mendelian conditions collectively affect 8% life births; however, less than half of these patients receive a molecular diagnosis and the pathophysiology of disease for many known causal variants remains elusive [1,2]. Therefore, new disease-associated genes and causal variant discoveries need to be coupled with adequate disease modeling to understand the pathophysiology of the studied conditions and to develop effective treatments. Animal models and cell lines have been used for decades to understand human biology; however, the development of technologies to obtain human-derived induced pluripotent stem cells (hiPSCs) revolutionized the field of human biology [3,4,5,6,7,8,9,10]. This is particularly true for disease modeling in tissues that cannot be easily obtained from patients, such as the retina. The most prevalent group of Mendelian diseases of the retina is known as the inherited retinal degenerations (IRDs). IRDs are important causes of severe vision impairment or blindness affecting more than 2 million people worldwide and are caused by mutations in one of ~300 genes [11,12,13]. Due to the high genetic heterogenicity of IRDs, the disease mechanism for many of its forms is still unclear, especially for genes that have ubiquitous expression but a very specific retinal phenotype, such as the splicing factor genes, *PRPF3* and *PRPF8* [14,15], or *NMNAT1*, which encodes Nicotinamide Mononucleotide Adenylyltransferase 1, an enzyme crucial for biosynthesis of NAD+ (Nicotinamide Adenine Dinucleotide) in the cell nucleus [16,17,18,19].

Patient-derived hiPSCs represent a reliable resource for studying retinal diseases, but hiPSC line accessibility is limited, and new line derivation is costly and not always successful. Alternatively, isogenic disease models in established hiPSC lines can be generated using prime editing (PE), which can target up to 90% of the known pathogenic variants [20]. The clonal capacity of the hiPSCs makes them ideal for generating such models since cells carrying the desired edit can be selected based on their genotype and in the absence of off-target mutations [21,22,23]. Monoclonal cultures of PE-hiPSCs can be expanded and differentiated into 3D retinal organoid culture systems [24,25,26,27,28,29,30]. hiPSC-derived 3D retinal organoids have been used extensively to model retinal development [31,32,33,34,35,36] and to investigate disease mechanisms of inherited retinal degenerations (IRDs) [23,25,37,38,39,40] and can be employed for subsequent validation of gene therapies [41,42].

PE is a versatile cutting-edge CRISPR-based tool for genome engineering useful for introducing precise genetic variants into the organism or cell line genomes for disease modeling [43,44,45,46,47,48,49]. Compared to other DNA repair systems like homology-directed repair (HDR), PE technology can introduce point mutations, small insertions, and deletions into the genome with great precision and minimal off-target effects or unwanted insertions and deletions (indels) [20,50]. PE is especially valuable for editing hiPSCs, maximizing the in-depth analysis of pathophysiological mechanisms associated with specific genetic variants across isogenic lines [43,44,46,47] in a context that would be difficult to replicate using animal models [51]. Nevertheless, a low editing efficiency can hinder its use in hiPSCs as these cells are difficult to transfect, and PE efficiency is impaired by their efficient mismatch repair mechanism (MMR) [52,53,54,55,56]. Additionally, in some cases, the genetic context may restrict the screening of multiple PE guide RNAs (pegRNAs) suggested by the software, as these may bind too far from the target site, reducing their efficiency for genome modification. In such situations, alternative strategies are required to achieve the desired genetic edits [52,57].

In this study, we aimed to develop a PE platform to efficiently edit hiPSCs to generate isogenic models for three genetic forms of IRDs: (1) early-onset autosomal recessive (ar) IRD caused by a homozygous c.25G>A (p.V9M) mutation in the *NMNAT1* gene, (2) autosomal dominant (ad) IRD caused by a heterozygous c.1481C>T (p.T494M) mutation in *PRPF3,* and (3) adIRD caused by a heterozygous c.6926A>C (p.H2309P) mutation in *PRPF8* gene. To do so, we optimized the PE workflow to edit hiPSCs without manipulating key PE components but instead using a combinatorial strategy of plasmid concentrations and PE component ratios. We found that our optimized system enabled efficient generation of isogenic models of IRDs with PE efficiencies of up to 25% within 5 weeks, including cell banking and a thorough post-editing QC.

## 2. Results

### 2.1. Target Selection

To test the PE technology in hiPSCs with relevance to retinal disease, we chose three IRD genes with ubiquitous expression but associated with an isolated retinal phenotype *NMNAT1*, *PRPF3*, and *PRPF8* (Supplementary Table S1) [14,15,16]. As the first target, we chose *NMNAT1*, in which bi-allelic pathogenic variants lead to early-onset retinal degeneration [16,58,59,60]. *NMNAT1* encodes Nicotinamide Mononucleotide Adenylyltransferase 1, an enzyme crucial for the biosynthesis of NAD+ (Nicotinamide Adenine Dinucleotide) in the cell nucleus. NAD+ is a vital coenzyme involved in numerous metabolic processes, including DNA repair and cellular energy production [17,61]. Yet, despite its essential role, *NMNAT1* mutations cause retinal degeneration exclusively. As a first approach to disease modeling using PE, we specifically targeted one of the most common missense mutations present in the *NMNAT1*-IRD patients, the c.25G>A in exon 2, leading to p.V9M substitution.

As the second and third PE targets, we chose *PRPF3* and *PRPF8* associated with autosomal dominant (ad) forms of retinitis pigmentosa (RP) [15,62]. PRPF3 and PRPF8 are highly conserved proteins playing a role in pre-mRNA splicing as components of the U4/U6-U5 tri-snRNP complex [63,64,65,66]. Despite being expressed ubiquitously, the mechanisms by which mutations in these genes cause an exclusively retinal phenotype remain unclear [38,66,67]. We chose to model the *PRPF3* c.1482C>T (p.T494M) and the *PRPF8* c.6926A>C (p.H2309P) mutations, which are recurrent pathogenic variants in these genes [14,15,62,63,68,69,70,71]. Disease onset for the patients carrying *PRPF3* and *PRPF8* mutations is variable, and it may start as early as in the first decade of life [71,72]. However, mice heterozygous for the *Prpf3* c.1482C>T (p.T494M) and the *Prpf8* c.6926A>C (p.H2309P) mutations show late-onset phenotype with aberrant RPE morphology at 24 months and decreased retinal function in the *Prpf3* model only [14]. The discrepancy between the mouse and human data suggests important interspecies differences, and therefore, modeling these diseases in hiPSC-derived RPE and retinal organoids offers an opportunity to better understand the pathophysiology of the splicing factors associated with IRD.

### 2.2. PE Strategy

To generate the isogenic models of IRDs with PE harboring the selected variants (Supplementary Table S1), we selected hiPSC lines based on the absence of alteration in the genomic regions of interest as determined by WGS or WES. We used our fully characterized OGli001 hiPSC line [68], the established hiPSC lines IMR90-4 [73] and a SIX6-p2A-GFP/POU4F2-p2A-tdTomato dual reporter on the same IMR90-4 background [74,75].

**Figure 1:**
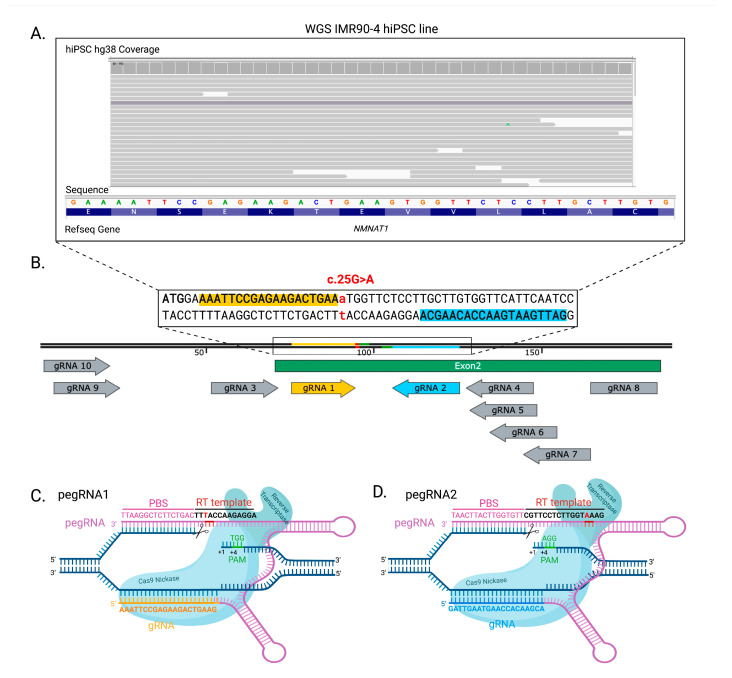
PE3 prime editing strategy to generate the isogenic model for an *NMNAT1* c.25G>A (p.V9M) mutation. (**A**). WGS analysis of the genome of the hiPSC line IMR90-clone 4 depicts no variants in the region of interest for *NMNAT1* editing compared to the reference human genome hg38. (**B**). Two gRNAs out of ten were selected for molecular cloning based on the distance to the desired edit position (**C**). pegRNA1 and (**D**). pegRNA2 anneal to the complementary DNA strand. Cas9 Nickase nicks the opposite strand, allowing for PBS annealing and reverse transcription along the RT template that incorporates the desired single nucleotide edit (red letter).

To design the PE strategies, in silico analysis of the genomic region of interest was performed. For the pegRNA design directed toward the *NMNAT1* p.V9M mutation, we selected a 200 bp sequence surrounding the c.25G>A variant and used PegFinder to select the closest pegRNAs and secondary nicking gRNAs to the region of interest. From ten candidates, we selected two pegRNAs, with nicking sites 3 and 14 nucleotides to the desired edits (pegRNA1 and pegRNA2, respectively) (Figure 1). The pegRNAs with the recommended primer binding sites (PBS) of 16 nucleotides and the recommended RT-templates of 13 and 17 nucleotides for pegRNA1 and pegRNA2, respectively, were cloned into a plasmid containing a U6 promoter [20] (Figure 1). Additionally, we selected two nicking gRNAs on opposite strands to the pegRNA recognition sequences (Supplementary Table S2). The addition of a nicking gRNA, known as PE3, is a common strategy to improve PE efficiency [20].

### 2.3. Validation of sgRNA

**Figure 2:**
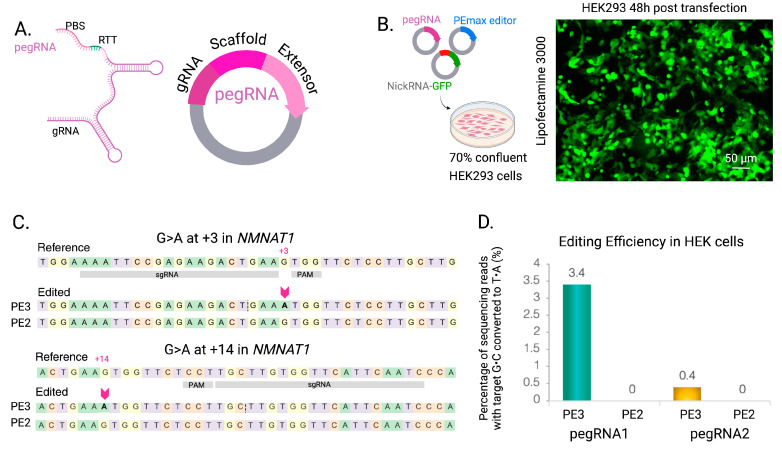
Validation of PE in HEK-293T cells. (**A**). Each pegRNA has 3 key components: a gRNA or spacer, a scaffold, and an extension with the PBS and the RTT sequence, which introduces the desired edit. (**B**). Confluent HEK cells were co-transfected using Lipofectamine 3000 with the pegRNA, the PEmax editor, and a nicking guide plasmid harboring a reporter EGFP cassette. Then, 48 h post-transfection, approximately 80–90% of the HEK cells expressed GFP. Scale bar = 50 μm. (**C**). Three days after plasmid delivery, genomic DNA was extracted, and PE was analyzed by NGS. pegRNA1 was designed to introduce a G>A edit at +3 position from the nicking site in *NMNAT1,* and pegRNA2 was designed to introduce the same edit at +14 position from the nicking site. (**D**). Editing efficiencies were determined by NGS and expressed as the percentage of alleles with G•C target converted to T•A. NGS showed editing only occurred with PE3 with low efficiency: 3.40% for pegRNA1 and 0.42% for peg RNA2.

After molecular cloning, the PE2 (without the nicking gRNAs) and PE3 (with the nicking gRNA) strategies for PE *NMNAT1* p.V9M mutation were assessed in vitro in HEK-293T cells. In all cases, we used the PEmax editor (http://pegfinder.sidichenlab.org, accessed on 30 November 2024), which contains a human codon-optimized RT, and mutations in SpCas9, to enhance editing efficiency and improve nuclear localization [52]. We used a standard nicking gRNA plasmid containing a U6 promoter and [76] expressing EGFP under the CAG promoter that also served as a transfection efficiency control. We observed 80–90% of EGFP-positive HEK-293T cells 24–48 h post-transfection. To determine the PE efficiency, we performed next-generation sequencing (NGS) analysis and observed only PE3 with pegRNA1, and nicking gRNA1 showed a 3.40% of total reads with target G•C converted to T•A (1147 reads without indels). The remaining three combinations returned undetectable (PE2 pegRNA1 and PE2 pegRNA2 with 0%) or very low edits (PE3 pegRNA2 0.42%) (Figure 2).

**Figure 3:**
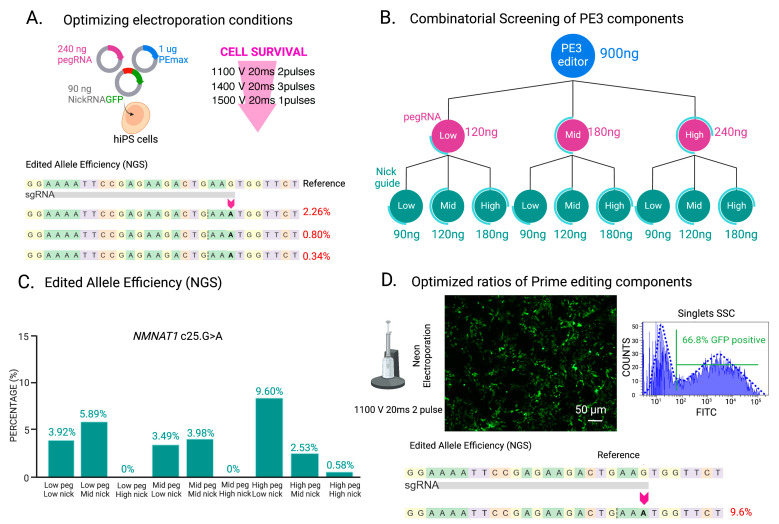
Optimization of PE of hiPSCs for *NMNAT1* c.25G>A. (**A**). Optimization of electroporation conditions for the efficient delivery of PE components in the cells using 1 μg of PEmax editor, 90 ng of nicking guide RNA, and 240 ng of pegRNA1. Three different electroporation parameters were tested. The best condition was 1100 V, 20 ms, and 2 pulses, resulting in the highest cell survival and the highest percentage of edited alleles detected by next-generation sequencing (NGS). (**B**). Combinatorial screening of different concentrations of PE components. Using the optimal electroporation parameters, a total of 9 different combinations of PE components were assessed to optimize PE efficiency. PEmax was set at 900 ng, and three doses of pegRNA (low, 120 ng; medium (mid), 180 ng; and high, 240 ng) and three doses of nicking guide RNA (low, 90 ng; medium (mid), 120 ng; and high 180 ng) were included. (**C**). NGS analysis of the bulk electroporated hiPSCs showed the maximum editing efficiency (9.60%) was achieved with high pegRNA1 and low nicking gRNA. (**D**). PE efficiency under optimized conditions demonstrated a substantial increase in the percentage of GFP-positive cells (66.80%) post-electroporation that translated into a notable increase in editing efficiency. Scale bar = 50 μm.

Encouraged by these results, we tested our working combination of PE3 with pegRNA1 and nicking guide1 in hiPSCs. Despite Lipofectamine™ being used in hiPSCs to deliver canonical CRISPR/Cas9 components, other methods, such as electroporation, have been shown to be more efficient in these cells [77,78] and have recently been used for PE in hiPSCs [47]. We used electroporation as a delivery method in the hiPSCs following parameters previously described [52], with cell densities and PE component ratios extrapolated from the HEK-293T cell experiments. We observed that post-electroporation, less than 1.00% of hiPSC colonies expressed EGFP-positive cells, and as expected, the NGS results showed a low percentage of edited alleles (0.67%, 352,218 reads without indels) (Supplementary Figure S1) compared to that observed on HEK-293T cells. We hypothesized that it was due to inefficient delivery conditions. Therefore, we tested three additional electroporation conditions and increased the pegRNA plasmid concentration to 240 ng (high pegRNA Table 1). Under the same total DNA amount, 1100 V, 20 ms, and 2 pulses yielded the best cell survival rate and highest editing (Figure 3A). Other electroporation protocols specifically reported to be effective in hiPSCs using the Neon electroporation system [79] were also tested, but no edited alleles were detected, or 100% of the cells died after electroporation.

### 2.4. Combinatorial Screening of PE Components to Optimize PE of hiPSCs

After establishing optimal Neon electroporation conditions for hiPSCs that resulted in a higher cell survival rate, we observed that the editing efficiency, as assessed by NGS, remained low, at only 2.30% of edited alleles (Figure 3A). To address this issue, we conducted a combinatorial analysis to explore various ratios of PE components, aiming to identify conditions that would enhance the efficiency of the desired genetic edit. Specifically, we focused on pegRNA1 to introduce the c.25G>A mutation in the *NMNAT1* gene. Concerned about the potential negative impact of high total DNA amounts on cell viability after electroporation, we reduced the PEmax plasmid dose to 900 ng for subsequent optimization steps, as this plasmid is the largest of the three plasmids used. The PEmax editor was combined with three different doses of pegRNA1, categorized as low (120 ng), medium (180 ng), and high (240 ng), alongside three doses of the nicking gRNA, which we designated as low (90 ng), medium (120 ng), and high (180 ng), giving nine possible combinations (Figure 3B, Table 1). The editing efficiency was subsequently determined using amplicon sequencing on the bulk of electroporated cells (Figure 3C). The highest editing efficiency observed across all conditions was 9.60% when the high dose of pegRNA1 and a low dose of the nicking gRNA1 was utilized. Conversely, using the high pegRNA1 dose in combination with mid and high doses of the nicking guide resulted in decreased editing efficiencies of 2.53% and 0.58%, respectively (Figure 3C). These findings suggest that the total amount of DNA is a critical factor influencing editing outcomes, likely due to its direct correlation with cell survival post-electroporation.

**Figure 4:**
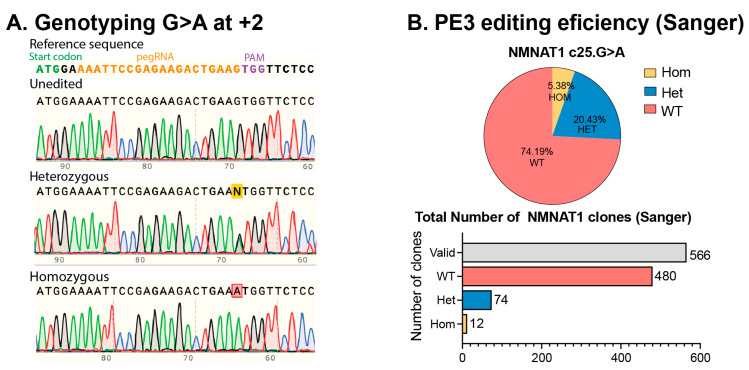
Generation of *NMNAT1*^V9M/V9M^ isogenic clones. (**A**). Genotype confirmation and (**B**). determination of the PE efficiency for *NMNAT1* c.25 G>A using Sanger sequencing. PE3 efficiency accounted for over 25.00% of edited clones. Data in (**A**) are representative of *n* ≥ 2 independent replicates.

Our optimized ratios of PE components in hiPSCs demonstrated a transfection efficiency exceeding 60% of GFP-positive cells post-electroporation, as assessed by fluorescent microscopy and FACS, with NGS identifying 9.60% of correctly edited alleles for the c.25G>A *NMNAT1* mutation (Figure 3D). Following single clonal expansion of the *NMNAT1* lines, we identified 5.38% homozygous p.V9M *NMNAT1* clones and 20.43% heterozygous p.V9M *NMNAT1* clones, with an overall editing efficiency of over 25.00% by Sanger sequencing (Figure 4A,B).

### 2.5. Generation of the PRPF3 c.1482C>T (p.T494M) and PRPF8 c.6926A>C (p.H2309P) Isogenic Lines

Using the optimized electroporation parameters and plasmid concentrations, we prioritized the PE2 system over PE3 due to its lower cytotoxicity and higher cell survival rates post-electroporation. Following this approach, we generated two additional isogenic models of IRDs with the c.1482C>T (T494M) mutation in *PRPF3* and the c.6926A>C (H2309P) mutation in *PRPF8.* The PE2 system demonstrated sufficient efficiency to achieve the clinically relevant heterozygous genotype for both mutations, eliminating the need to proceed with PE3.

**Figure 5:**
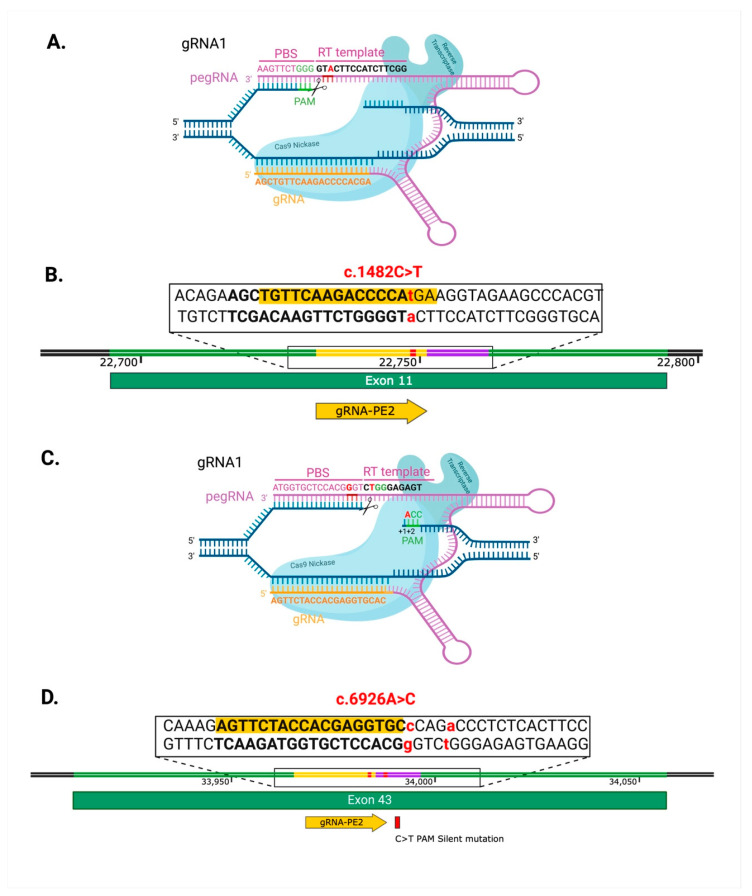
Prime editing strategy to generate the isogenic model for *PRPF3* c.1482C>T (p.T494M) mutation and for *PRPF8* c.6926A>C (p.H2309P) mutation. (**A**). The gRNA1 anneals with the complementary DNA strand. Cas9 Nickase nicks the PAM-containing strand of the target DNA. The PBS anneals with the PAM-containing strand, and RTase extends the 3′ end using the RT template. (**B**). pegRNA was selected for molecular cloning based on the distance to the mutation and sequence length. (**C**). The gRNA1 anneals with the complementary DNA strand. Cas9 Nickase nicks the PAM-containing strand of the target DNA. The PBS anneals with the PAM-containing strand, and RTase extends the 3′ end using the RT template. (**D**). pegRNA was selected for molecular cloning based on the distance to the mutation and sequence length.

**Figure 6:**
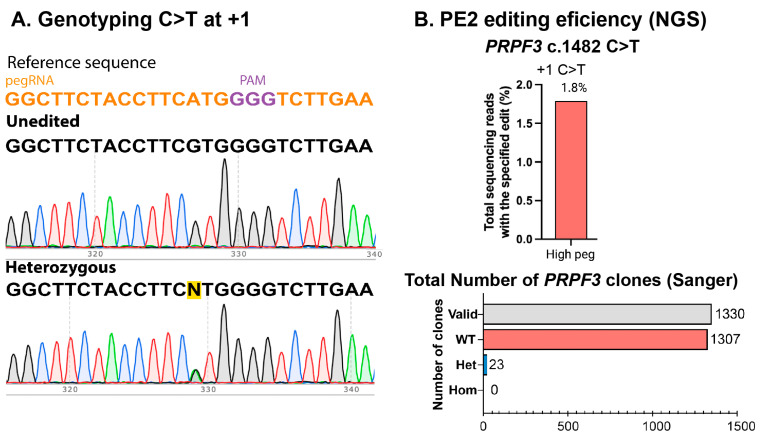
Generation of *PRPF3* isogenic clones. (**A**). Genotype confirmation and (**B**). determination of the PE efficiency with high pegRNA for *PRPF3* c.1482 C>T (p.T494M) using NGS and Sanger sequencing. Data in (**A**) are representative of *n* ≥ 2 independent replicates.

**Figure 7:**
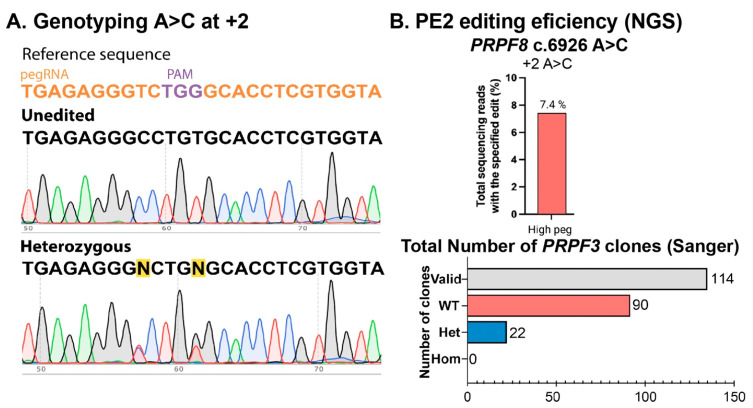
Generation of *PRPF8* isogenic clones. (**A**). Genotype confirmation and (**B**). determination of the PE efficiency with high and maximal pegRNAs for *PRPF8* c.6926 A>C (p.H2309P) using NGS and Sanger sequencing. Data in (**A**) are representative of *n* ≥ 2 independent replicates.

For pegRNA selection, we used pegFinder and a 360 bp sequence surrounding the *PRPF3* c.1482C>T variant in exon 11. We chose the optimal pegRNAs based on the proximity to the desired edit in position +1 with respect to the CRISPR/Cas9 cut position (Figure 5A,B) and the recommended primer binding sites (PBSs) of 13 nucleotides and RT-template of 14 nucleotides (Table 2 and Table S3) [20]. For the *PRPF8* c.6926A>C (p.H2309P) isogenic line, we used a 300 bp sequence surrounding the variant within the *PRPF8* exon 43 genomic region and selected the pegRNA most proximal to the desired edit in position +2 with respect to the CRISPR/Cas9 cut position (Figure 5C,D, Table 3). Additionally, we introduced a silent mutation in the PAM to improve the editing efficiency and chose the recommended PBS of 13 nucleotides and RT-template of 14 nucleotides [20]. The optimized conditions yielded 1.8% editing efficiency in *PRPF3* and 7.40% editing efficiency in *PRPF8*, all of which were heterozygous, as seen after genotyping of individual clones (Figure 6 and Figure 7).

### 2.6. Expansion and Characterization of Single Clones

**Figure 8:**
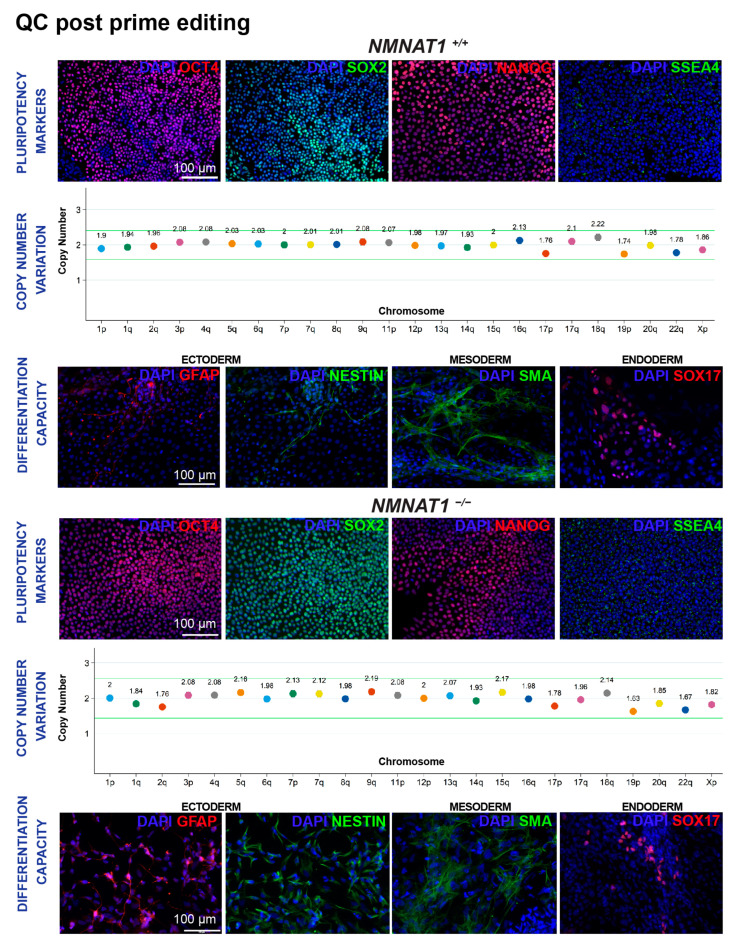
Characterization of hiPSC wild-type *NMNAT1^+/+^*, and homozygous *NMNAT1*^−/−^ clones showing immunofluorescence (IF) images of SOX2^+^, SSEA4^+^, OCT^+^, and NANOG^+^ cells for pluripotency markers; embryoid bodies exhibiting NESTIN^+^ and GFAP^+^ (ectoderm), SMA^+^ (mesoderm), and SOX17^+^ (endoderm) for germ layer makers; and chromosomal copy number variation analysis. Representative images from *n* > 5. Scale bar = 100 μm.

The colony morphology of the edited hiPSC *NMNAT1*, *PRPF3*, and *PRPF8* clones was assessed by bright-field microscopy. Edited hiPSC colonies retained the prominent nucleoli and high nuclear-to-cytoplasmic ratio typically observed in the undifferentiated state of this cell type even after several passages (Supplementary Figure S2). As part of our QC pipeline, we characterized the edited *NMNAT1, PRPF3,* and *PRPF8* hiPSC lines for the expression of pluripotency markers (SOX2^+^, SSEA4^+^, OCT4^+^, and NANOG^+^ cells) and assessed their differentiation capacity towards the three germ layers using the embryoid body (EB) model. All the edited hiPSC lines could form EBs, and when plated, the cells deriving radially away from the aggregates expressed markers of ectoderm (GFAP^+^ and NESTIN^+^), endoderm (SOX17^+^), and mesoderm (SMA^+^) cells as determined by immunofluorescence (IF). As hiPSCs often acquire recurrent genetic abnormalities during prolonged cultures [80,81,82] and these karyotypic abnormalities could alter the stemness and impact the differentiation, we analyzed the edited clones for chromosomal abnormalities, determining the copy number variation (Figure 8, Figures S3 and S4). Edited lines without a trisomy were expanded and cryopreserved following established protocols.

### 2.7. Off-Target Analysis

Given recent concerns regarding potential off-target effects associated with PE [83,84,85], we performed in silico and in vitro off-target analyses as part of our QC. Using platforms such as Cas-OFFinder and Off-Spotter enables the identification of overlapping genomic regions bearing high sequence homology to the sites of the PE and nicking gRNAs. We validated the absence of the top off-targets by Sanger sequencing (Supplementary Figure S5). This validation is of major importance since potential genotoxic effects, including by-products such as deletions and translocations, have been described for PE and base editing in hiPSCs [86]. Another approach that has been reported in the literature is a genome-wide strategy named PE-tag, which is based on the insertion of an amplification tag at PE sites [83].

## 3. Discussion

Isogenic disease models allow researchers to perform an in-depth investigation of the pathophysiological mechanisms of disease without interference from other possibly contributing variants that may be present in the patient-derived disease models [87]. Although patient-derived genetic disease models have been widely implemented for studying disease mechanisms and drug efficacy, the genetic differences between control and patient cells can lead to inaccuracies in the result interpretations [87]. The underlying genetic background of a model may harbor additional variants that contribute to the disease and challenge the phenotype rescue. For instance, attempts to restore vision in rd1 mice using AAV-mediated *Pde6b* gene supplementation were unsuccessful until a second mutation in *Gpr179* affecting bipolar cells was identified as responsible for an altered b-wave [88]. Therefore, limitations of patient-derived models due to genetic background discrepancies and the scarcity of patient samples [87,89,90] underscore the relevance of implementing isogenic hiPSC models.

**Figure 9:**
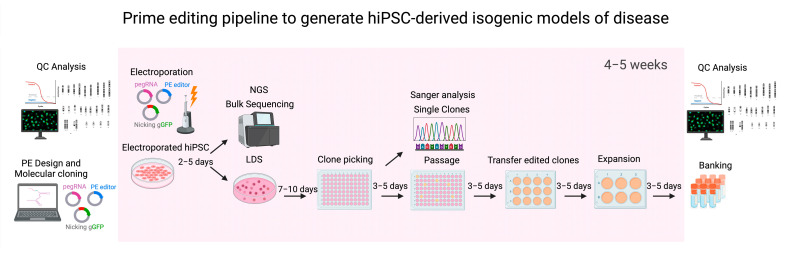
Prime editing (PE) pipeline. Our PE pipeline includes the in silico design of the pegRNA components and molecular cloning to ensemble the pegRNAs, the nicking, and the PEmax editor plasmids. We also performed a full QC analysis of the hiPSC lines prior to editing, including evaluating pluripotency and chromosomal abnormalities and assessing the differentiation capacity. Electroporation with different combinations of PE components was performed on highly confluent cultures to generate isogenic models for the *NMNAT1*, *PRPF3*, and *PRPF8* mutations. After 2–5 days, electroporated hiPSC lines were dissociated into single cells and passaged at low-density seeding (LDS). In parallel, NGS was performed to confirm editing has occurred in the bulk cultures and estimate the number of clones that need to be recovered from the LDS cultures. Single colonies from LDS cultures were manually dissected and passaged by mechanical disruption into 96-well plates, where the desired edit was confirmed through Sanger sequencing analysis. Confirmed edited clones were expanded first to 12-well plates and then to 6-well plates for banking. Cryopreserved seed and master banks were obtained in parallel to the QC analysis of the selected clones.

The great potential of hiPSCs, coupled with the advances in genome editing, has made them an ideal ex vivo system for disease modeling. However, to fully leverage the capabilities of PE for disease modeling and other clinical applications, it is essential to improve the methodologies for modifying hiPSCs. Therefore, we established a PE pipeline for the efficient generation of isogenic hiPSC lines (Figure 9). In our pipeline, we have focused primarily on the efficient delivery of PE components to the hiPSCs, which in the future can be coupled with further advances in the pegRNA design, manipulation of cellular DNA repair pathways, expansion of targeting scope, and enhancement of PE delivery methods in hiPSCs [91]. Our optimized PE workflow for hiPSCs has successfully generated 53 isogenic lines of IRDs, including 15 NMNAT1 p.V9M clones, 14 PRPF3 p.T494M, and 24 for *PRPF8* p.H2309P clones. Our approach focused on optimizing critical factors related to efficient transfection and cell viability, which involved electroporation parameters, cell count, total DNA amounts transfected, and ratios of the PE components. This strategy proved to be as effective as other studies using RNA delivery to improve transfection efficiencies in hiPSCs [47,52,92], underscoring that improving delivery can be as important as pegRNA optimization for efficient PE of hiPSCs. Once the electroporation parameters and the best working combination of PE key component ratios are determined, our established PE pipeline can efficiently generate an isogenic hiPSC line in 4–5 weeks, encompassing the design and molecular cloning of pegRNAs, electroporation of plasmids, selection and expansion of single clones, genotype confirmation by Sanger sequencing, and banking of isogenic lines (Figure 9).

### 3.1. Electroporation and Cell Viability

We have found that electroporation parameters set at 1100 V, 20 ms, and 2 pulses while keeping the total DNA amount under 1000 ng were optimal for the survival of all the selected hiPSCs. These conditions led to the highest transfection efficiencies and subsequently increased the PE outcomes. Moreover, under the same experimental parameters, we observed that PE3 enhanced the editing efficiency compared to PE2 only when cell viability post-electroporation was not compromised by excessive DNA cargo. To reduce the cargo, the pegRNA and the nicking sgRNA could be cloned in smaller plasmids, or the recently developed All-in-One PE plasmid consisting of three cassettes for expression of all PE3 components [93] could be used at the expense of losing versatility.

### 3.2. QC Analysis and Differentiation Capacity

A comprehensive QC analysis of the hiPSCs pre-and post-editing has proven crucial for efficiently editing different lines. This includes an imaging analysis of colony morphology, an immunofluorescence analysis to determine high levels of expression of pluripotency markers, and a copy number variation analysis to detect any potential chromosomal abnormalities. These abnormalities are often acquired by hiPSCs during prolonged cultures, especially when they are passaged as single cells [68]. As recurrent abnormalities in a hiPSC-edited clone may enhance its proliferation or reduce its differentiation capacity, making it genetically unstable [94], we assessed the differentiation capacity using the EB model on every clone as part of our QC.

Our gene editing workflow offers a powerful and safer alternative to traditional systems like HDR genome editing by minimizing off-target effects and avoiding the generation of double-strand breaks, which are associated with chromosomal rearrangements and other genotoxic risks commonly observed with HDR [20]. PE is believed to enable precise editing with minimal off-targets. However, there is an inherent risk of generating unwanted indel by-products due to the introduction of a nick in the non-edited strand when using the PE3 system [55]. This risk could be mitigated by implementing PE3b in hiPSCs, which also uses a nicking sgRNA but only targets the edited strand, preventing the nicking of the non-edited DNA strand and reducing the likelihood of unwanted indels. To rule out potential off-target effects associated with PE, we use in silico tools like Cas-OFFinder and Off-Spotter to identify genomic regions with high sequence homology to the PE target sites and those of the nicking guide RNAs and validate the absence of the off-targets with the highest homology through PCR, gel electrophoresis, and Sanger sequencing. This validation is crucial given the risk of genotoxic effects, such as deletions and translocations, reported in hiPSCs edited by PE and base editing. To our knowledge, this is the first PE validation to combine in silico and in vitro off-target analyses, whereas other strategies often rely solely on predictive scores.

### 3.3. PE Key Component Ratios

Using PEmax as an editor, we optimized suboptimal pegRNAs to efficiently edit hiPSCs using both PE2 and PE3 strategies. Once the electroporation parameters and delivery protocols were established, we closely screened the ratios of the PE components to identify combinations that yielded the highest PE efficiency. For the c.25G>A *NMNAT1* edit, we tested in parallel the three main PE components, including the PEmax, pegRNA, and nicking sgRNA. Our combinatorial screening revealed that the most effective weight ratio was 10:2.7:1 of PEmax, pegRNA, and nicking sg RNA plasmids. Notably, increasing the nicking guide did not yield further improvements, likely due to increased cell death associated with excessive DNA delivery.

The generation of isogenic cell line models for *PRPF3* and *PRPF8*, achieving editing efficiencies up to 7.00% as determined with NGS, did not require the additional nicking gRNA needed for *NMNAT1*^V9M/V9M^. We concluded that a higher editing efficiency is needed to edit both alleles and generate homozygous lines. After several rounds of optimization of electroporation parameters and combinations of PE component ratios, a 9.60% editing efficiency was achieved by NGS, and this translated to over 25.00% of edited clones harboring the *NMNAT1* V9M mutation as determined by Sanger. The improvements observed with PE3 reaffirm the finding that the overall efficiency of PE in hiPSCs could be enhanced by stimulating the de novo synthesized 3′ flap to be used as a repair template instead of the original 5′ flap [52,95].

Our work suggests that careful optimization of electroporation conditions, control of cell viability, a comprehensive QC prior to editing, and the combinatorial analysis of the ratios of PE components can successfully install edits in hiPSCs to generate isogenic models of disease.

## 4. Materials and Methods

### 4.1. pegRNA Design and In Silico Analysis

Wild-type sequences for *NMNAT1* (chr1:9,972,019–9,972,189), *PRPF3* (chr1:150,344,162–150,344,261), and *PRPF8* (chr17:1,651,108–1,651,310) at the region of interest were obtained from the University of California Santa Cruz Genome Browser (https://genome.ucsc.edu). Exon sequences were obtained and processed on SnapGene^®^ (version 7.1.2). pegFinder pegRNA designer for CRISPR prime editing (http://pegfinder.sidichenlab.org) was used to design PE and nicking guides for installing a V9M mutation in *NMNAT1*, T494M in *PRPF3*, and H2309P in *PRPF8*. Wild-type/reference and edited sequences were used as input, and PEmax was the chosen editor (Supplementary Table S2). In the case of V9M *NMANT1* PE, ten pegRNAs were designed (Table 4 and Table 5) and analyzed to predict off-targets and nicking efficiency using CRISPOR. In the case of *PRPF3* and *PRPF8*, candidate sequences are shown in Table 2 and Table 3, respectively.

### 4.2. Molecular Cloning

Standard molecular cloning procedures were performed following established protocols [96]. The plasmid used to express the pegRNAs was obtained from Addgene (Watertown, MA, USA) (pU6-pegRNA-GG-acceptor #132777). pU6-peg-GG-acceptor was digested using BsaI-HF^®^ v2 restriction enzyme to clone the pegRNAs as described in Anzalone et al., 2019 [20]. NEBridge^®^ Golden Gate Assembly Kit (New England Biolabs, Ipswich, MA, USA, NEB E1601) was used following the manufacturer’s protocol. PEmax-expressing plasmid (pCMV-PEmax Addgene #174820) [52] was used as a prime editor. An additional nicking gRNA expressing a GFP cassette was cloned into a U6-gRNA-CAG-GFP vector obtained from Dr. Qin Liu’s lab. This was used to introduce an additional nick on the opposite DNA strand and increase the efficiency of PE. A list of generated plasmids and synthetic gRNA/ngRNA/pegRNA sequences is provided in Supplementary Tables S2 and S3, respectively.

### 4.3. Culture and Transfection of HEK-293T Cells

HEK-293T cells (ATCC CRL-3216) were maintained in DMEM (Thermo Fisher Scientific 11965092, Waltham, MA, USA) supplemented with 15% FB Essence (Avantor 10799-390, Radnor Township, PA, USA), 2 mM glutamine (Thermo Fisher Scientific 25030081), 1 mM nonessential amino acids (Thermo Fisher Scientific 11140050), and 1 × Penicillin-Streptomycin (Thermo Fisher Scientific 10378016) and passaged every other day with 0.25% Trypsin with Ethylenediaminetetraacetic acid (EDTA) (Thermo Fisher Scientific 25300054). One day before transfection, the cells were seeded into 0.2% gelatin-coated 12-well plates at 1 × 10^4^ cell/cm^2^ to allow for 80% confluency the following day. HEK cells were co-transfected following the Lipofectamine 3000 protocol in opti-MEM (Thermo Fisher Scientific) with 500 ng of pCMV-PEmax (Addgene #174820) [20], 330 ng of pU6-pegRNA (Addgene #132777) [52], and 170 ng of an additional nicking gRNA (JL65_pFYF_BsmBI EX16_gRNA1, gift from Qin Liu’s lab) [76] for the PE3 strategy or 500 ng of pCMV-PEmax and 500 ng of pU6-pegRNA for the PE2 strategy. The cells were collected for genomic DNA extraction and NGS-based allele quantification three days post-transfection.

### 4.4. Culture and Maintenance of hiPSCs

IMR90-4 human iPSCs (hiPSCs) were purchased from WiCell Research Institute, Inc. (Madison, WI, USA, WISCi004-A). This cell line was generated through the viral transduction of IMR90 fetal lung fibroblasts with a combination of the OCT4, SOX2, NANOG, and LIN28 genes [73]. SIX6-p2A-GFP/POU4F2-p2A-tdTomato dual reporter hiPSCs on the same IMR90-4 background were developed by Karl Wahlin from the University of California, San Diego [74,97]. The OGIi0001A hiPSC line was derived and fully characterized from a healthy male donor in our lab [68]. The absence of variations in the genome of the hiPSC lines used in this study was corroborated using whole genome sequencing (WGS) or whole exome sequencing (WES). All hiPSC lines were routinely maintained on growth factor-reduced (GFR) Matrigel basement membrane matrix (Corning 354230, Corning, NY, USA)-coated 6-well plates (Corning 353046) in mTeSR™ Plus (STEMCELL Technologies 100-0276, Vancouver, BC, USA) medium and incubated at 5% CO_2_ in a 37 °C humidified incubator.

### 4.5. Passage as Cell Aggregates

Cells at ~70% confluence in 6-well plates were passaged using 1 mL of ReLeSR™ (STEMCELL Technologies 100-0484) per well. ReLeSR™ was removed within 1 min, and the cells were incubated at 37 °C for 4 min. Gentle tapping was performed to detach the cells. hiPSCs were resuspended in 2 mL/well of mTeSR™ Plus, and the aggregate mixture was pipetted up and down twice to ensure the breakup of large aggregates. The ideal aggregate size was 50–200 μm, confirmed under a microscope. The cell aggregate mixture was plated at 1:30 density.

### 4.6. Passage as Single Cells

Cells at ~90% confluence were passaged using 1 mL/well of StemPro™ Accutase™ Cell Dissociation Reagent (Gibco A1110501, Waltham, MA, USA) to obtain a single-cell suspension. The cells were incubated at 37 °C for 4 min with StemPro™ Accutase™, which was inactivated with 1:10 dilution in mTeSR™ Plus. The cells were centrifuged at 1000× *g* rpm for 4 min at RT The supernatant was removed, and the cells were resuspended in mTeSR™ Plus. hiPSCs were counted using a Neubauer counting chamber (Hausser Scientific HS3120, Horsham, PA, USA), and cell concentration was adjusted and plated at the desired density in mTeSR™ Plus supplemented with 10 µM ROCK-inhibitor (STEMCELL Technologies 72305). This type of passage was used for electroporation and low-density seeding cultures.

### 4.7. Electroporation of hiPSCs

Electroporation was performed following the manufacturer’s protocol using a Neon™ electroporation system (Invitrogen MPK5000, Waltham, MA, USA), a Neon™ Transfection System Pipette Station (MPS100), a Neon™ Transfection System Pipette (Invitrogen MPP100), and a Neon™ Transfection System 10 μL Kit (Invitrogen MPK1096). hiPSCs at 90% confluence were dissociated as single cells, as described above. The cells were resuspended in mTeSR™ Plus and counted using a Neubauer counting chamber (Hausser Scientific HS3120), and concentration was adjusted to 2 × 10^4^ cells/μL. hiPSCs were centrifuged at 1000× *g* rpm for 4 min, and the pellet was resuspended in 11 μL of buffer R and mixed with all the different plasmid combinations described in Table 1. Three different electroporation parameters were used: 1100 V (pulse voltage), 20 ms (pulse width), and 2 pulses; 1400 V, 20 ms, and 3 pulses; 1500 V, 20 ms, and 1 pulse. Electroporated hiPSCs were transferred into GFR Matrigel-coated 24-well plates containing 3 mL of mTeSR™ Plus supplemented with 10µM ROCK-inhibitor (STEMCELL Technologies 72305) without antibiotics. Cell morphology was monitored every day after this point.

### 4.8. Bulk NGS and Allele Quantification

hiPSCs were maintained on GFR Matrigel-coated 24-well plates in triplicates for 48–72 h to monitor the GFP-expressing cells. One of the samples was used for bulk NGS-based allele quantification. DNA was extracted using QuickExtract™ DNA Extraction Solution (Biosearch Technologies QE09050, Hoddesdon, UK) and quantified using a NanoDrop One Spectrophotometer (Thermo Scientific 13-400-518). DNA was mutation-region-amplified by PCR using the primers designed with Primer3 Plus platform to target the region of interest (Table 6). The targeted region was amplified using Phusion High-Fidelity PCR Master Mix (Thermo Scientific F531L). The PCR product was purified using the column-based ZYMO DNA Clean & Concentrator-5 PCR Purification 5 μg Partial Kit (ZYMO Research D4004, Irvine, CA, USA). Samples were sequenced at 500 K reads with a sequencing length of 2 × 150 bp in an Illumina (San Diego, CA, USA) sequencer at the Genomics Core of the Ocular Genomics Institute (OGI) of Massachusetts Eye and Ear Infirmary, Harvard Medical School. Editing was considered positive when the number of reads aligned exceeded 90%, mutant allele frequency > 70%, and indels frequency < 5%. CRISPResso2 [98] was used to analyze the editing frequency expressed as the percentage of edited alleles of each group.

### 4.9. Clonal Selection by Low-Density Seeding

hiPSCs at confluence ~70% were dissociated as single cells, as described in the previous section. Low-density seeding was achieved by plating 500 cells into a 60 mm Nunc™ Petri Dish (Thermo Fisher 150326) in 3 mL mTeSR™ Plus supplemented with 10% CloneR2 (STEMCELL Technologies 100-0691). Single colonies were picked after 7–10 days and expanded in Falcon^®^ 96-well Clear Flat Bottom plates (Corning 353072). Once hiPSCs reached a confluence of ~70%, they were passaged and expanded for banking. The remaining cells in the 96-well plate were collected by Sanger sequencing.

### 4.10. Sanger Sequencing

DNA was extracted from hiPSC clones in QuickExtract™ DNA Extraction following the manufacturer’s protocol. Regions of interest were amplified with Phusion™ High-Fidelity DNA Polymerase (Thermo Scientific F-530XL) using the primers listed in Table 6, and synthesized by Integrated DNA Technologies. The PCR product was purified with ExoSAP-IT™ Express PCR Product Cleanup Reagent (Applied Biosystems 75001.4X.1.ML, Waltham, MA, USA) following the manufacturer’s protocol. Forward or reverse primer was added to each purified PCR product in a 1:40 dilution. Samples were submitted for Sanger sequencing to the Genomics Core of the Ocular Genomics Institute at Mass Eye and Ear Infirmary, Harvard Medical School Teaching Hospital. Sanger sequencing was analyzed on SnapGene^®^ (Dotmatics Limited, Boston, MA, USA, version 7.1.2).

### 4.11. Banking and QC of hiPSC-Edited Clones

Clones at a confluence of ~70% were washed with DPBS and lifted using ReLeSR™ as described above. Cells from 6-well plates were centrifuged at 1000× *g* rpm for 4 min and resuspended in 3 mL/well of CryoStor^®^ CS10 (BioLife Solutions 07930, Bothel, WA, USA). An amount of 1 mL of cell suspension was aliquoted per Cryogenic Vial (Corning 431417) for cryopreservation and seed banking. We have recently described the full characterization and QC our hiPSC lines undergo [68]. The same thorough QC analysis was performed on the edited clones resulting from this study, which we describe briefly in the following sections.

#### 4.11.1. Pluripotency Assessment

Retention of stemness by the edited clones was determined by the expression of pluripotency markers, including transcription factors NANOG, OCT4, and SOX2, and the cell surface marker SSEA4 [99,100]. Briefly, subconfluent hiPSC cultures (60% confluence) in 24-well plates were fixed with freshly prepared 4% Paraformaldehyde Aqueous Solution (Electron Microscopy Sciences 15714, Hatfield, PA, USA) at 4 °C for 10 min. Post-fixation, the samples were washed with 0.5 mL of DPBS, and the plates were sealed with Parafilm™ (Bemis PM999, Sheboygan Falls, WI, USA) and stored at 4 °C until the time of the immunofluorescence analysis, described below.

#### 4.11.2. Spontaneous Differentiation Capacity/Embryoid Body (EB) Model

The differentiation capacity of the edited clones was evaluated using the embryoid model (EB) [101]. For EB formation, hiPSCs at confluence ~90% were dissociated using 1 mL/well of StemPro™ Accutase™. The single cells were resuspended in mTeSR™ Plus with 10 µM ROCK-Inhibitor at a concentration of 1000 cells per 100 μL per well in a clear, round-bottom, ultra-low-attachment 96-well plate (Corning 7007) to allow EB aggregation for three days. Half-medium changes (aspirating 50 μL and adding fresh 50 μL) were performed every two days with EB medium from days 3 to 6 (Table 7). On day 7 of differentiation in suspension, 24 EBs were collected with Transfer pipettes (Thermo Fisher 14-563-4789) and plated into a GFR-Matrigel-coated 24-well plate (Corning 353047). Fetal Bovine Serum (10%, Gibco A5209502) was added to the EB medium formulation for the first 24h to promote attachment (Table 7). On day 14, the medium was removed, and the EBs were washed with DPBS (Gibco 14190144) at room temperature (RT) and fixed with freshly prepared 4% Paraformaldehyde Aqueous Solution (Electron Microscopy Sciences 15714) at 4 °C for 10 min. Post-fixation, plates with the EBs were washed with 0.5 mL of DPBS per well and sealed with Parafilm™ (Bemis PM999). Plates can be stored at 4 °C until immunofluorescence analysis for all three germ layers, as described below. The markers used for characterization include NESTIN and GFAP (ectoderm), SOX17 (endoderm), and SMA (mesoderm).

#### 4.11.3. Chromosomal Abnormalities

DNA was extracted from hiPSC samples using the QIAamp DNA Blood Mini Kit (Qiagen 51104, Hilden, Germany). DNA concentration was measured on a Nanodrop™ One Spectrophotometer (Thermo Scientific 13-400-525). The minimum DNA concentration was 50 ng/µL. All samples (50 µL per sample) were analyzed by Stem Genomics Inc. (Durham, NC, USA) using their proprietary iCS-Digital™ PSC Technology. No chromosomal abnormalities were found in any of the selected hiPSCs.

#### 4.11.4. Mycoplasma Test

All hiPSC lines and differentiated cell cultures used for this project were routinely tested for mycoplasma detection using the MycoAlert^®^ Mycoplasma Detection Kit (Lonza LT07-418, Basel, Switzerland) based on the manufacturer’s instructions.

#### 4.11.5. Immunofluorescence

The plates were washed 3X 5 min with 1X PBS (Invitrogen AM9625) followed by 2X 5 min washes with 0.1% Tween^®^ 20 (Sigma-Aldrich P1379, St. Louis, MO, USA) in PBS solution. Permeabilization was performed for 30 min using 0.1% Tween^®^ 20 and 0.5% Triton™ (Sigma-Aldrich ×100) in PBS solution. The samples were blocked for 30 min using 0.2% gelatin from cold water fish skin (Sigma-Aldrich G7041) containing 0.25% Triton™ in PBS solution. Primary antibodies (Table 8) were prepared in 1 mL blocking solution, and the samples were incubated overnight at 4 °C. The next day, the samples were washed 3× for 10 min with 0.1% Tween^®^ 20 in PBS solution. Secondary antibodies (Table 9) were prepared in 1 mL of blocking solution, along with 1 μg/mL of DAPI (Sigma-Aldrich D9542). and incubated for 1 h in the dark at RT. Following this point, samples were kept in the dark and washed 3× for 5 min with 0.1% Tween^®^ 20 in PBS solution, followed by one wash for 5 min with PBS. The samples were dehydrated in 100% ethanol (Pharmco 111000200CSGL, Hallandale Beach, FL, USA) for 1 min and allowed to dry. VECTASHIELD Vibrance^®^ Antifade Mounting Medium (Vector Laboratories H-1700, Newark, CA, USA) was used as a mounting medium, and coverslips (Fisher Scientific NC9950471) were placed, avoiding any bubbles. Samples were stored at 4 °C in the dark. A fluorescent microscope (Nikon Eclipse Ti, Tokyo, Japan) was used to capture images (20×). Immunofluorescence experiments were conducted with more than 5 biological replicates to ensure robustness and reproducibility of the results.

### 4.12. Off-Target Analysis

We performed the off-target analysis using two online platforms, Cas-OFFinder (http://www.rgenome.net/cas-offinder/, accessed on 30 November 2024) and Off-Spotter (https://cm.jefferson.edu/Off-Spotter/, accessed on 30 November 2024), to identify overlapping genomic regions bearing high sequence homology to the sites of the PE and nicking gRNAs. Cas-OFFinder, described in Bae et al., 2014 [102], compiles all the 23 bp DNA sequences comprising 20 bp sequences corresponding to the sgRNA sequence of interest 5′-NRG-3′ PAM and then compares it with the query sequence counting the mismatch number. Then, Off-Spotter, which was developed by Pliatsika et al., 2015 [103], identifies all genomic sites that satisfy the PAM constraint and are identical or nearly identical to the given sgRNA and a PAM by building tables with pre-computed results that are compared with the query sequence. Both methods recognize the genomic sites that match the candidate gRNA sequence with a given PAM. Hence, common output results allow for thorough validation of the in silico screening. Query sequences were allowed to a maximum of three mismatches (Table 10). The top 10 sequences exhibiting the highest sequence homology were selected based on the following criteria: mismatch number up to three and locus of hit located in a gene involved in retinal development. The primers flanking the *PPP1R42* genomic region in *PRPR3E* WT and HET, and *ELP4* in *NMNAT1* WT and Hom were designed using Primer3 plus tool for off-target sequencing analysis, as shown in Table 10.

For validation of the absence of any off-targets in the selected clones, Sanger sequencing was performed. DNA extraction was performed by using a Qiagen DNeasy mini kit (Qiagen 69504) according to the manufacture’s protocol. The extracted DNA was quantified by using the Nanodrop method for DNA quantification. We also performed in silico PCR by using the UCSC In-Silico PCR tool to check the specificity of our designed PCR primers prior to performing PCR. The amplified products were cleaned by using the ZYMO DNA Clean & Concentrator-5 PCR Purification 5ug Partial Kit (ZYMO Research D4004). The purified products were checked for amplification of the targeted region by using 5ul of amplified product on 1.2% agarose gel. An amount of 100 ng of the amplified product was sent for Sanger sequencing with the forward primer of each amplicon to the Genomics Core of the Ocular Genomics Institute at Mass Eye and Ear Infirmary, Harvard Medical School Teaching Hospital. SnapGene^®^ (version 7.1.2) was used to analyze the sequencing results (Supplementary Figure S5).

## 5. Conclusions

This study focused on the development of isogenic hiPSC models as an alternative approach for disease modeling compared to patient-derived models. These isogenic models might mitigate challenges such as genetic variability and additional mutations present in patient-derived models, ensuring greater accuracy in mimicking diseases.

Combining hiPSCs and precision genome editing technologies like PE, we have developed an efficient platform to generate isogenic lines in 4–5 weeks. This pipeline involves optimizing key factors, such as electroporation parameters and the ratio of PE components, that can be fine-tuned to maximize editing efficiency without compromising cell viability. Our strategy allowed for the generation of several isogenic lines for IRD modeling, including the heritable p.V9M mutation, which is causative of *NMNAT1*-associated Leber congenital amaurosis, and the p.T494M mutation in *PRPF3* and the p.H2309P mutation in *PRPF8*, both associated with retinitis pigmentosa. However, our system can be adapted to generate models of other Mendelian diseases.

QC and genotyping validations are integral to this process. QC measures include monitoring colony morphology, pluripotency markers, chromosomal stability, and differentiation capacity. Additionally, an in silico and in vitro combined analysis was proposed to determine the minimal off-target effects and indel by-products inherent to PE techniques. Optimized ratios of PE components demonstrated significant improvements in editing efficiency, achieving up to 25% with some mutations.

This work underscores the necessity of combining methodical optimization with robust QC to enhance the efficiency and reliability of isogenic hiPSC-based disease models, which hold immense potential for studying disease mechanisms and developing targeted therapies.

## Figures and Tables

**Table 1:** Plasmid combination concentration tested for electroporation.

hiPSCs	Low Peg	Low Peg, Medium Nick	Low Peg, High Nick	Medium Peg	Medium Peg, Medium Nick	Medium Peg, High nick	High Peg	High Peg, Medium Nick	High Peg, High Nick
pegRNA (ng)	120	120	120	180	180	180	240	240	240
Nicking guide (ng)	90	120	180	90	120	180	90	120	180
Editor (ng)	900	900	900	900	900	900	900	900	900

**Table 2:** Candidate primary editing sgRNAs for *PRPF3*.

sgRNA Sequence	Orientation	Distance to Edit Start
AGCTGTTCAAGACCCCACGA	sense	+1
CGTGGGCTTCTACCTTCGTG	antisense	−0
ACGTGGGCTTCTACCTTCGT	antisense	−1
GACGTGGGCTTCTACCTTCG	antisense	−2
TGCCATCTGAGCTCTGACGT	antisense	−17
TTGCCATCTGAGCTCTGACG	antisense	−18
TCTAATTTGATGCGAGTATT	sense	+29
GAGGTTTCCACCCAATCCCA	antisense	−58
TTGGGAAAGGTACTTGCCCG	antisense	−77
CCCCCAGAAGAGTTTGGGAA	antisense	−90
TGGACCCCCCAGAAGAGTTT	antisense	−95
ATGGACCCCCCAGAAGAGTT	antisense	−96
CTCTCCCCTCCCTCCAAATA	antisense	−115
CTGAGGGAAAGAGAGAGCAA	antisense	−142

**Table 3:** Candidate primary editing sgRNAs for *PRPF8*.

sgRNA Sequence	Orientation	Distance to Edit Start	PAM Edit
AGTTCTACCACGAGGTGCAC	sense	+2	1 (G>A)
GCAAAGTTGAGGAAGTGAGA	antisense	−6	0
AGCAAAGTTGAGGAAGTGAG	antisense	−7	0
CCCCAAAGAGTTCTACCACG	sense	+9	0
CCTGCAGGAGAGCAAAGTTG	antisense	−17	0
AGTAAACCTCCCCCTCCTGC	antisense	−32	0
CATGAAATATGAGCTACAGC	sense	+36	0
TCCTTCTCCCCGAAGGTGTT	sense	+70	0
AGGGAAACGGTCAGGCATAC	antisense	−71	0
GGCTGTTTCCTTCTCCCCGA	sense	+77	0

**Table 4:** Candidate primary editing sgRNAs for *NMNAT1*.

sgRNA Sequence	Orientation	Distance to Edit Start
AAATTCCGAGAAGACTGAAG	sense	+3
GATTGAATGAACCACAAGCA	antisense	−14
ACAACTTCAAGTTCTTACCA	sense	+26
CTGAGGTGCATGTTGGTGAT	antisense	−35
CCTGAGGTGCATGTTGGTGA	antisense	−36
AAACAACCTGAGGTGCATGT	antisense	−42
TGGCCAGCTCAAACAACCTG	antisense	−52
TGTTCCATTCATGTAGTCCT	antisense	−72
TCCTTTGTAGACAACAAGGG	sense	+73
TTTTCCTTTGTAGACAACAA	sense	+76

**Table 5:** Candidate secondary nicking sgRNAs (PE3) for *NMNAT1*.

Nicking sgRNA Sequence	Orientation	Distance to pegRNA Nick
TGGCCAGCTCAAACAACCTG	antisense	−55
AAACAACCTGAGGTGCATGT	antisense	−45
TGTTCCATTCATGTAGTCCT	antisense	−75
CTTGAAGTTGTTGATCTAAA	antisense	−47
ACCTCCCTTGTTGTCTACAA	antisense	−81

**Table 6:** Primer design for NGS to target region of interest for *NMNAT1, PRPF3*, and *PRPF8*.

Gene	Forward Primer	Reverse Primer
*NMNAT1*	ACAACAAGGGAGGTGTCACAG	GACTACATGAATGGAACAGGTA
*PRPF3*	CACTAGATGCTGTGATCAGTGTG	GGAAGTTTGGCCCAGTATGGA
*PRPF8*	GCCCTGTTAACATTGGCTGTT	AGGGAAACGGTCAGGCATAC

**Table 7:** EB media formulation for hiPSCs.

Days 3 to 6 (250 mL)	Days 7 to 14 (250 mL)
DMEM/F12/GlutaMAX™ 196.75 mL (Life Technologies Corporation; Grand Island, NY, USA)	DMEM/F12/GlutaMAX™ 171.75 mL
Penicillin-Streptomycin (0.1%) 0.25 mL (Sigma-Aldrich; Saint Louis, MO, USA)	Penicillin-Streptomycin (0.1%) 0.25 mL
KnockOut™ Serum Replacement (KOSR) (20%) 50 mL (Life Technologies Corporation; Grand Island, NY, USA)	KOSR (20%) 50 mL
Minimum Essential Medium (MEM) Non-essential Amino Acids (NEAA) (1×) 2.5 mL (Life Technologies Corporation; Grand Island, NY, USA)	MEM NEAA (1X) 2.5 mL
2-Mercaptoethanol (50 mM) 0.5 mL (Life Technologies Corporation; Grand Island, NY, USA)	2-Mercaptoethanol (50 mM) 0.5 mL
	Fetal Bovine Serum 25 mL (Life Technologies Corporation; Paisley, Scotland, UK)

**Table 8:** Primary antibody list.

Antibody	Species	Company	Cat. No.	Dilution
GFAP	Rabbit	Agilent Dako (Santa Clara, CA, USA)	Z0334	1 in 200
NANOG	Rabbit	Cell Signaling (Danvers, MA, USA)	4903S	1 in 200
NESTIN	Mouse	Novus Biologicals (Toronto, ON, USA)	NBP1-92717	1 in 500
OCT4	Mouse	Cell Signaling	75463S	1 in 250
SMA	Mouse	Agilent Dako	M0851	1 in 200
SOX17	Goat	R&D Systems (Minneapolis, MN, USA)	AF1924	1 in 500
SOX17	Rabbit	Abcam (Cambridge, UK)	ab224637	1 in 500
SOX2	Rabbit	Cell Signaling	3579S	1 in 400
SSEA4	Mouse	Cell Signaling	4755T	1 in 400

**Table 9:** Secondary antibody list.

Host	Against	Conjugate	Isotype	Company	Cat. No	Dilution
Goat	Rabbit	Alexa Fluor 488	IgG	Invitrogen	A11034	1 in 500
Goat	Rabbit	Alexa Fluor 555	IgG	Invitrogen	A21429	1 in 500
Goat	Mouse	Alexa Fluor 488	IgG	Invitrogen	A21121	1 in 500
Goat	Mouse	Alexa Fluor 555	IgG	Invitrogen	A21422	1 in 500
Donkey	Goat	Alexa Fluor 488	IgG	Invitrogen	A11055	1 in 500
Rabbit	Goat	Alexa Fluor 555	IgG	Invitrogen	A21431	1 in 500

**Table 10:** Off-target analysis and the primers used for the selected off-target sequencing.

Reference Sequence	Target Sequence	Gene	Hit DNA Sequence	Chromosome Position	Mismatches	Forward Primer	Reverse Primer
*NMNAT1*	TGGCCAGCTCAAACAACCTG	*GPR98*	AAATaCCGAGAAatCTGAAAGGG	chr5, −90646382	3		
		*ELP4*	AAATTagaAGAAGACTGAAAGGG	chr11, +90646382	3	5′ TCTGCGTTGTGAATGGGGTT 3′	5′ GCCCTGTTTGTTCAAGCACC 3′
		*DAB1*	AAAaTCaaAGAAGACTGAAATGG	chr1, −57325067	3		
		*NEGR1*	AAATTCtGAaAAGtCTGAAATGG	chr1, −71465063	3		
		*SPATA17*	AAATTCaGtGAAaACTGAAAAGG	chr1, +217793809	3		
		*LMNA*	GaACCTgAGGaTGTTTGAGCAGG	chr1, −156133617	3		
		*TSHR*	TGGCtAGtTCAAACAACCTGAGG	chr14, −81032599	2		
*PRPF3*	AGCTGTTCAAGACCCCACGANGG	*PPP1R42*	AGCTGTTCAAGACCCCACGAAGG	chr8, −66984739	0	5′ CAGCTCAATGCTCCAGTGACA 3′	5′ CCCCTTCCACTACTACCACGTT 3′
		*NEDD4L*	gGCTtTTCAAGACCCCACGgAGG	chr18, +58187120	3		

## Data Availability

The original contributions presented in this study are included in the article and Supplementary Materials. For any further inquiries, please contact the corresponding author.

## Supplementary Materials

The following supporting information can be downloaded at https://www.mdpi.com/article/10.3390/ijms26010114/s1.
