# Supplementary material for: Optimized Prime Editing of Human Induced Pluripotent Stem Cells to Efficiently Generate Isogenic Models of Mendelian Diseases
Source: Int J Mol Sci. 2024 Dec 26;26(1):114. doi: 10.3390/ijms26010114 (PMC11719581; doi:10.3390/ijms26010114)
Supplement: Supplementary file 1 [file ijms-26-00114-s001.zip › ijms-3385740-supplementary.pdf]

Supplementary materials
Cerna-Chavez et al ., Optimized Prime Editing of hiPSCs to Efficiently Generate Isogenic Models of Mendelian Diseases.

Table of Contents

***Supplementary materials* 1**

***Supplementary tables* 1**

Supplementary Table S1. Selected IRD-associated gene variants for prime editing 1

Supplementary Table S2. List of plasmids used in this study 1

Supplementary Table S3. Synthetic PegRNA key components used in this study 2

***Supplementary Figures* 2**

Supplementary Figure S1. Validation of PE in hiPSCs to edit c.25G>A NMNAT1 mutation.................. 2

Supplementary Figure S2. Colony Morphology. 3

Supplementary Figure S3. Characterization of PRPF3 hiPSCs.. 4

Supplementary Figure S4. Characterization of PRPF8 hiPSCs.. 5

Supplementary Figure S5. Off-target analysis for PRPF3 and NMNAT1. 6

Supplementary tables

Supplementary Table S1. Selected IRD-associated gene variants for prime editing.

Candidate gene	Variant/ Substitution	Inheritance/ Genotype	Associated Phenotype	Chr. Position	Allele Change	Biotype
NMNAT1	c.25G>A (p.V9M)	arIRD	Leber congenital amaurosis (LCA)	chr1:9,972,019-9,972,189	G>A	coding
PRPF3	c.1481C>T (p.T494M)	adIRD	Retinitis Pigmentosa (RP)	chr1:150,344,162-150,344,261	C>T	coding
PRPF8	c.6926A>C (p.H2309P)	adIRD	Retinitis Pigmentosa (RP)	chr17:1,651,108-1,651,310	A>C	coding

ar: autosomal recessive. ad: autosomal dominant. IRD: inherited retinal disease.

Supplementary Table S2. List of plasmids used in this study.

plasmid	plasmid name	Origin/Reference	Addgene #
pegRNA	pU6-pegRNA-GG-acceptor	Anzalone *et al.*, 2019	132777
Editor	pCMV-PEmax	Chen *et al.*, 2021	174820
Nicking RNA guide	JL65_pFYF_BsmBI EX16_gRNA1	Gift from Qin Liu's lab	NA

pegRNA: prime editing guide RNA.

Supplementary Table S3. Synthetic pegRNA key components used in this study.

Gene to Edit	Desired edit	gRNA sequences	PAM	Sense 3' extension
NMNAT1	c.25 G>A (p.V9M)	AAATTCCGAGAAGACTGAA G	TGG	AGGAGAACCATTTCAGTCTTCTCGGAATT
NMNAT1	c.25 G>A (p.V9M)	GATTGAATGAACCACAAGC A	AGG	GAAATGGTTCTCCTTGCTTGTGGTTCATTCAAT
PRPF3	c.1481C>T (p.T494M)	AGCTGTTCAAGACCCCACGA	AGG	GGCTTCTACCTTCATGGGGTCTTGAAC
PRPF8	c.6926A>C (p.H2309P)	AGTTCTACCACGAGGTGCAC	ACC	TGAGAGGGTCTGGGCACCTCGTGGTAG

gRNA: guide RNA. PAM: protospacer adjacent motif.

Supplementary Figures

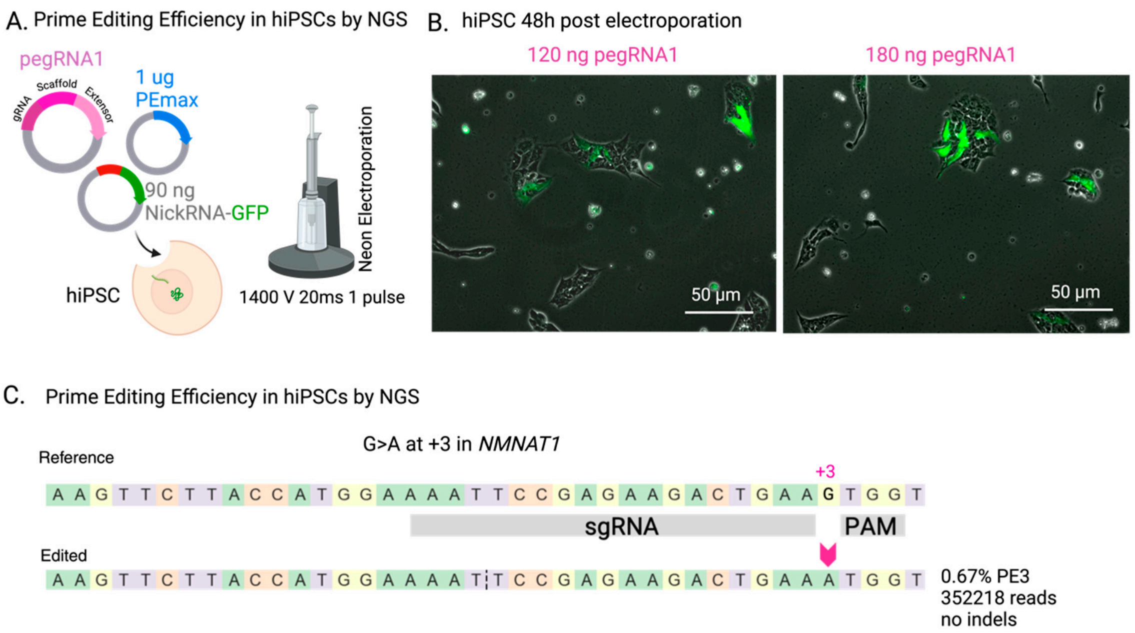

Supplementary Figure S1. Validation of PE in hiPSCs to edit c.25G>A NMNAT1 mutation.

A. 85,000 hiPSCs were electroporated with 1µg of PEmax editor and two different concentrations of the pegRNA1 plasmid designated as low: 120 ng and medium: 180 ng of pegRNA1 and the same concentration on the nicking guide: 90 ng. Confluent hiPSCs were co-electroporated using the Neon electroporation system with plasmids with the PEmax editor (1µg), the nicking guide (90ng), and the pegRNA1 at 1400V 20ms 1 pulse. **B.** Two different doses of the pegRNA1 were used (120ng and 180ng), and 48 h post-electroporation, very few cells (less than 5%) were GFP-positive, corresponding with cells that have incorporated the nicking guide plasmid carrying a GFP reporter cassette. Scale bar = 50 µm **C.** Genomic DNA was extracted, and PE3 efficiency was analyzed by NGS and expressed as the percentage of alleles with G•C target converted to T•A. Only the 180ng pegRNA1 combination installed a G>A edit at +3 in *NMNAT1* with a very low PE efficiency of 0.62%.

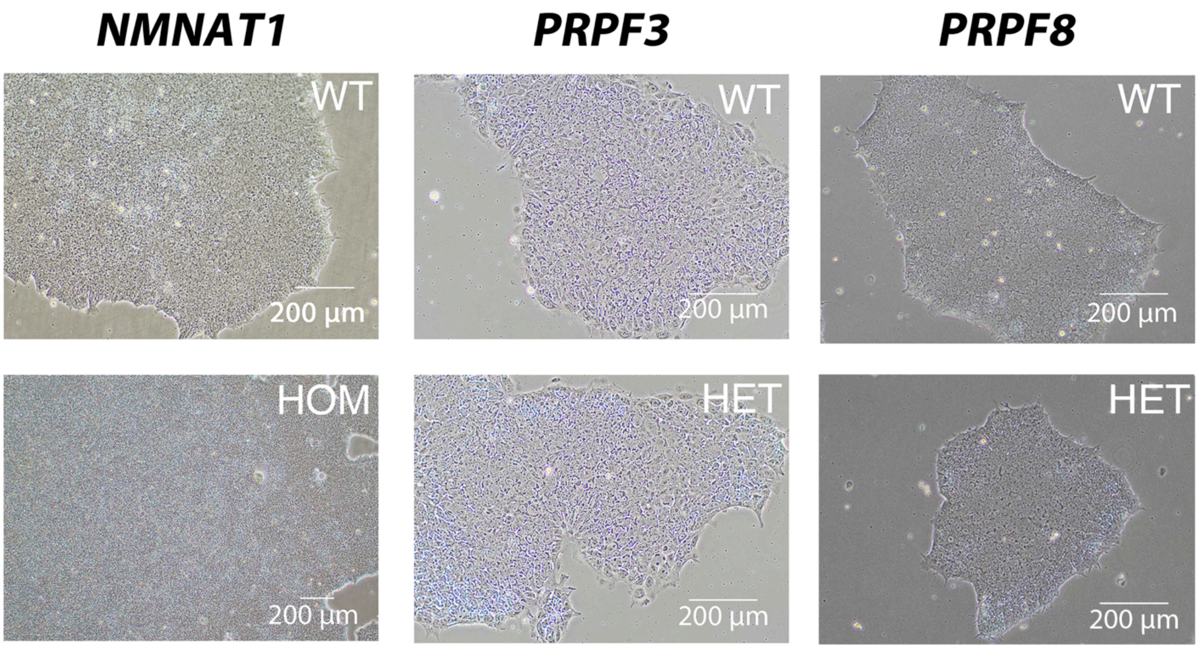

Supplementary Figure S2. Colony Morphology.
Bright-field images of single colonies of wild-type (WT), heterozygous (HET), and homozygous (HOM) hiPSC clones for the *NMNAT1, PRPF3,* and *PRPF8* lines showing tightly packed cells with the typical well-defined borders and a high large nucleus: cytoplasm ratio. Scale bar = 200 µm. Images are representative of n=10.

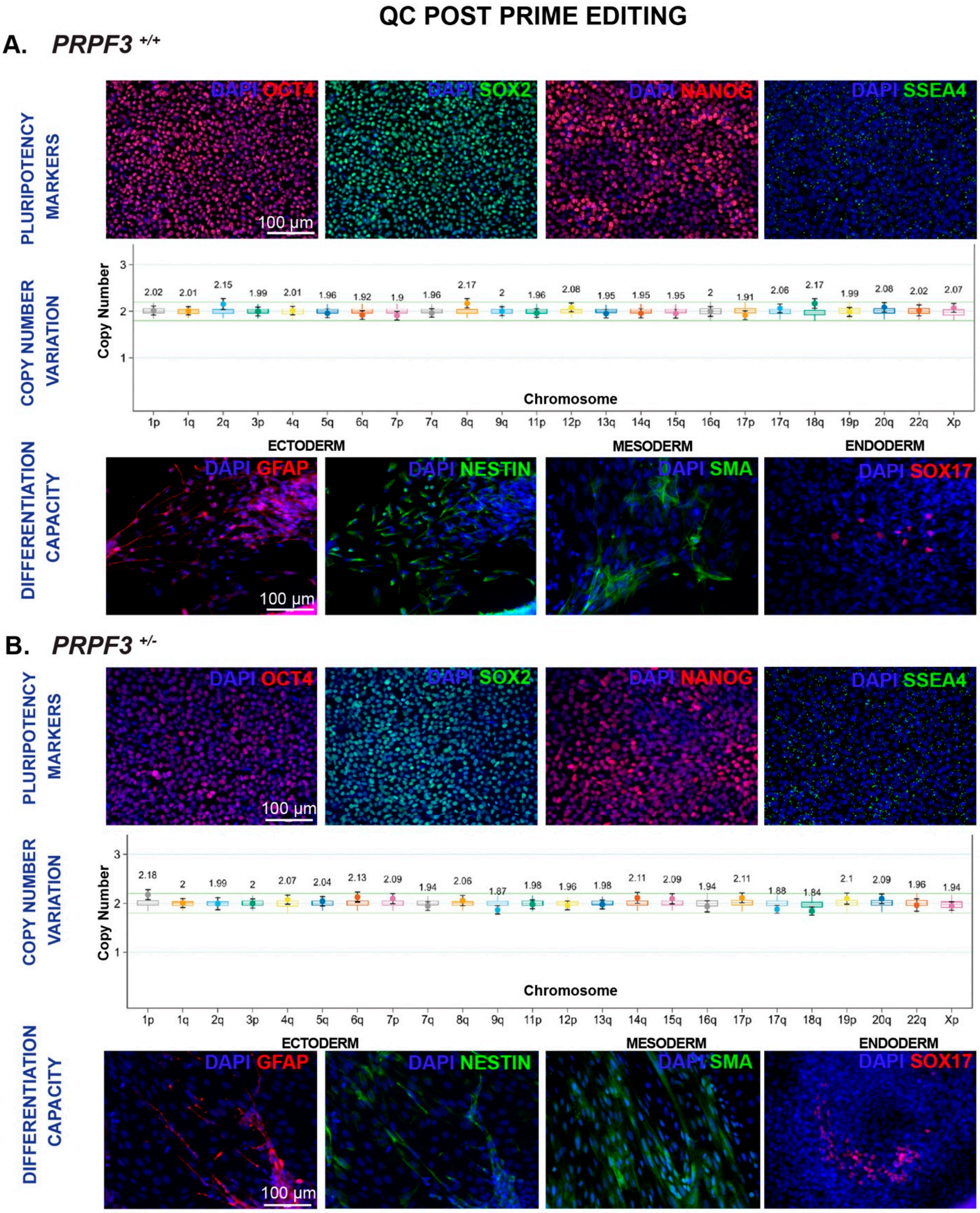

Supplementary Figure S3. Characterization of PRPF3 hiPSCs.

A. Wild-type *PRPF3*$^{+/+}$, and **B.** heterozygous *PRPF3*$^{+/-}$ clones showing IF images of SOX2^{+}, SSEA4^{+}, OCT4^{+}, and NANOG^{+} cells for pluripotency markers, embryoid bodies exhibiting NESTIN^{+}, GFAP^{+} (ectoderm), SMA^{+} (mesoderm), and SOX17^{+} (endoderm) for germ layer makers, and chromosomal copy number variation analysis. Images are representative of n>5. Scale bar = 100 µm.

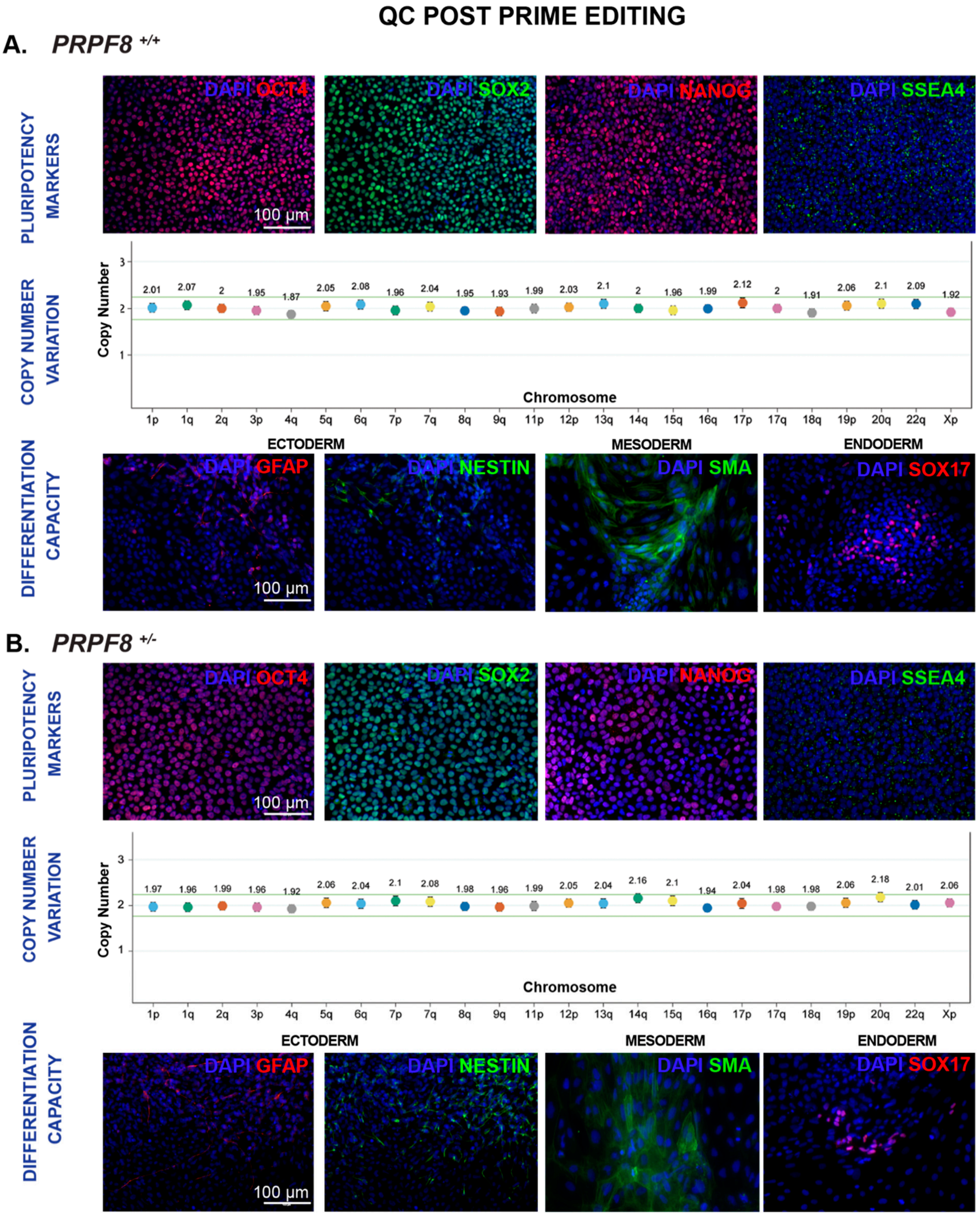

Supplementary Figure S4. Characterization of PRPF8 hiPSCs.

A. Wild-type *PRPF8*$^{+/+}$, and **B.** heterozygous *PRPF8*$^{+/-}$ clones showing IF images of SOX2^{+}, SSEA4^{+}, OCT4^{+}, and NANOG^{+} cells for pluripotency markers, embryoid bodies exhibiting NESTIN^{+}, GFAP^{+} (ectoderm), SMA^{+} (mesoderm), and SOX17^{+} (endoderm) for germ layer makers, and chromosomal copy number variation analysis. Images are representative of n>5. Scale bar = 100 µm.

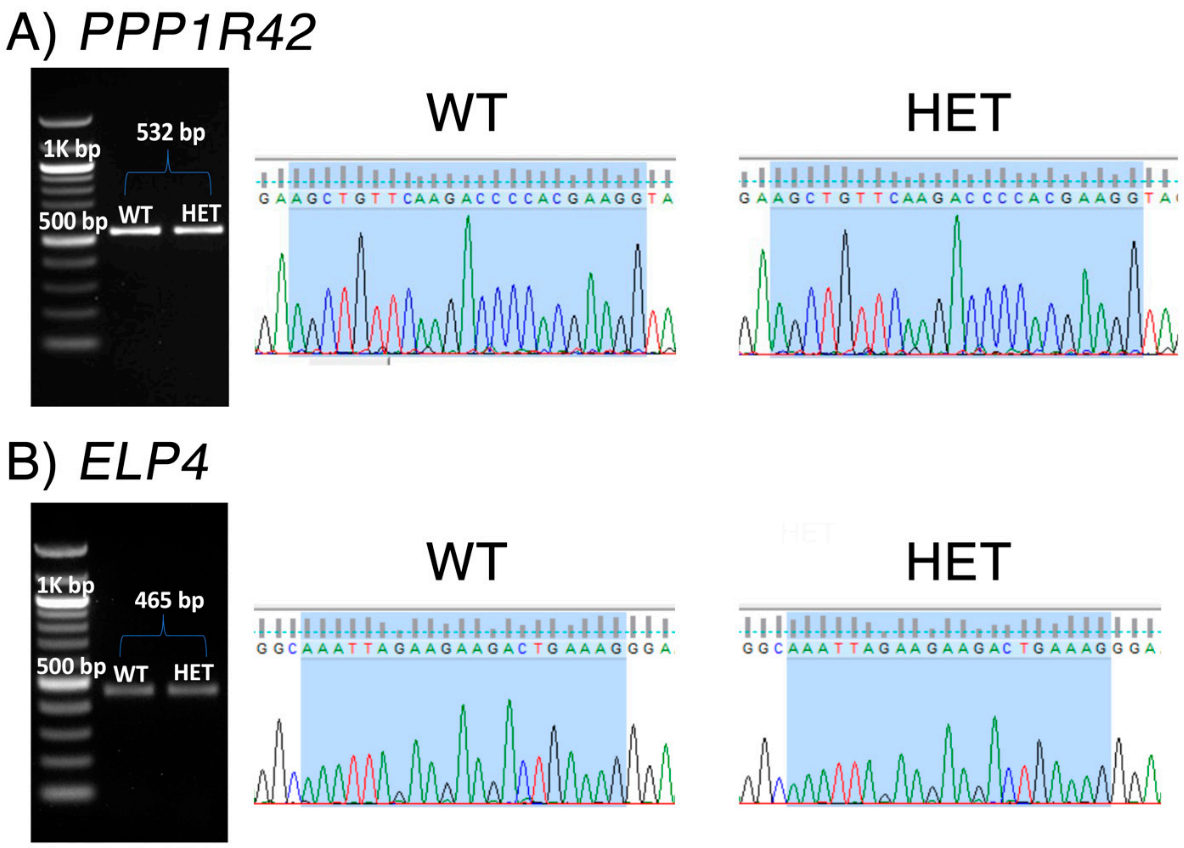

Supplementary Figure S5. Off-target analysis for PRPF3 and NMNAT1.
A. PCR product amplification for *PPP1R42* and Sanger sequencing chromatograms of the targeted region on *PRPF3* WT and HET. **B.** PCR product amplification for *ELP4* and Sanger sequencing chromatograms of the targeted region on *NMNAT1* WT and HET.
